# The implementation of inclusive education in South Africa: Reflections arising from a workshop for teachers and therapists to introduce Universal Design for Learning

**DOI:** 10.4102/ajod.v1i1.13

**Published:** 2012-11-13

**Authors:** Elizabeth M. Dalton, Judith Anne Mckenzie, Callista Kahonde

**Affiliations:** 1 TechACCESS Center, Rhode Island, USA; 2 Disability Studies Programme, School of Health and Rehabilitation Sciences, University of Cape Town, South Africa

## Abstract

South Africa has adopted an inclusive education policy in order to address barriers to learning in the education system. However, the implementation of this policy is hampered by the lack of teachers’ skills and knowledge in differentiating the curriculum to address a wide range of learning needs. In this paper we provided a background to inclusive education policy in South Africa and a brief exposition of an instructional design approach, Universal Design for Learning (UDL) that addresses a wide range of learning needs in a single classroom. We reported on a workshop conducted with teachers and therapists in South Africa as a first attempt to introduce UDL in this context. Knowledge of UDL was judged to be appropriate and useful by the course participants in the South African context as a strategy for curriculum differentiation in inclusive classrooms. Furthermore, knowledge of the UDL framework facilitates dialogue between teachers and therapists and provides a relatively simple and comprehensive approach for curriculum differentiation. We therefore conclude that there is potential for this approach that can be expanded through further teacher training.

## Introduction

It is a matter of grave concern that children with disabilities on the African continent face barriers in the education system for a multitude of reasons (ACPF 2011). In the South African context this has resulted in a massive exclusion of disabled children from education (Department of Education 2001). Despite the development of an inclusive education policy to address this exclusion, one of the issues that hampers progress is the lack of teacher skills in adapting the curriculum to meet a range of learning needs (Chataika, Mckenzie, Swart & Lyner-Cleophas 2012). This highlights the need for frameworks that empower teachers with the necessary skills to cater for learners with diverse needs. Universal Design for Learning (UDL) is one such framework that conceptualises and addresses the need for a more flexible curriculum designed to lower the barriers and to enable learners with widely varying needs to be included in the learning process (Brand, Favazza & Dalton 2012; Dalton 2005; Hall, Strangman & Meyer 2003).

In this paper we introduce UDL principles and implementation guidelines, and argue that UDL can have a useful application in the South African context of inclusive education. In order to make this claim, we present a background on inclusive education in South Africa, followed by a background and overview of UDL. We then present the report on a workshop on UDL conducted at the University of Cape Town in South Africa. We draw on evaluations made by the participants of the workshop in our discussion of the potential usefulness of UDL in their work contexts. Participant responses to specific activities are presented to illustrate their application of UDL in the workshop context. We conclude this paper with a discussion of the potential place of UDL in the implementation of inclusive education in South Africa.

## Inclusive education in South Africa

Since 1994, when democracy was established in South Africa, there has been a radical overhaul of government policy from an apartheid framework to providing services to all South Africans on an equitable basis. The provision of education for learners with disabilities has been part of that process and the development of an inclusive education system can be traced back to the nation’s founding document, the *Constitution of the Republic of South Africa, Act No. 108 of 1996* (Republic of South Africa 1996). In Section 29 (the Bill of Rights) it is stated that everyone has the right to ’a basic education, including basic adult education; and to further education, which the state through reasonable measures must make progressively available and accessible‘. It further states that the state may not discriminate directly or indirectly against anyone on one or more grounds, including disability.

The framework for an inclusive education system is laid out in *Education White Paper 6: Special Needs Education: Building an Inclusive Education and Training System* (Department of Education 2001). The scope of this policy is broad as it attempts to address the diverse needs of all learners who experience barriers to learning. The policy calls for a significant conceptual shift that is based on the following premises:

- all children, youth and adults have the potential to learn, given the necessary support
- the system’s inability to recognise and accommodate the diverse range of learning needs results in a breakdown of learning.

The policy asserts that in order to make inclusive education a reality, there needs to be a conceptual shift regarding the provision of support for learners who experience barriers to learning.

The Department of Basic Education has adopted a strategy that will drive the implementation of inclusive education policies. Summarised, this policy has two major components, elaborated in two sets of guidelines:

The National Strategy on Screening, Identification, Assessment and Support (SIAS); (Department of Education 2008) guides inclusive education policy by defining the process of identification, assessment, and enrolment of learners in special schools, and it curbs the unnecessary placement of learners in special schools. The SIAS strategy provides guidelines on early identification and support, the determination of nature and level of support required by learners, and identification of the best learning sites for support. The strategy also provides guidelines on the central role of parents and teachers in implementing the strategy.

The Guidelines for Responding to Learner Diversity in the Classroom through Curriculum and Assessment Policy Statements (Department of Education 2011) provide practical guidance to school managers and teachers on planning and teaching to meet the needs of a diverse range of learners. This document has recently been redrafted to incorporate curriculum changes in the Curriculum and Assessment Policy Statement (CAPS) and the revised document forms part of the CAPS orientation programme for teachers and education officials in the provinces.

Despite the enabling policy described above, the implementation of inclusive education in South Africa is slow and only partial (Wildeman & Nomdo 2007) The reasons for this are numerous and relate to problems that affect the education system as a whole, the role of special schools, and other support structures and conditions of poverty, amongst others (Stofile & Green 2006; Engelbrecht 2006). The issue that this paper addresses, however, is UDL as a potential framework to deal with teachers’ lack of knowledge and skills on how to design and present the curriculum in ways that can meet the diverse needs of learners in their classrooms. Teacher training programmes do not appear to be adequately addressing this need, resulting in stress for teachers and lack of progress of learners with disabilities (Engelbrecht, Swart, Eloff, 2001; Chataika *et al*. 2012; Engelbrecht 2006). The issue of curriculum differentiation is fundamental to the implementation of inclusion. In its apparent absence, children who experience barriers to learning cannot expect to have their needs met in a least-restrictive and inclusive setting with their age-mates. It was with this motivation that the conceptual and instructional framework of UDL was presented in a workshop held at the University of Cape Town in July 2011. Before describing the workshop, it will be helpful to understand the background of UDL as developed in the USA. The origins, rationale, research base, basic components and overall structure of UDL are given below.

### Educational history, inclusion, and universal design for learning

Over the past 37 years, the United States experienced significant changes in its system of education for students with disabilities. Prior to 1975, little attention was paid to meeting the needs of students with disabilities within a general education environment. Following the implementation of US Public Law 94–142 (Education for All Handicapped Children Act 1975), students have been included increasingly in the general education system and are increasingly expected to achieve in similar ways (and to similar standards) as their general education peers, thus supporting students with disabilities to be involved with their non-disabled peers to the maximum extent possible.

After a while, however, this system came into question as being insufficiently inclusive (Reynolds, Wang & Walberg 1987). A movement to fully include students with disabilities in US general education classrooms was the result (Fuchs & Fuchs 1994). With increasing access for learners with widely-varying needs, educational models were developed, going beyond mere accommodations and modifications, toward addressing *all* students’ educational needs through innovative and pro-active instructional design of the general education curriculum (Hitchcock, Meyer, Rose & Jackson 2002; Simmons & Kame’enui 1996). US schools are now responsible for providing effective instruction for all children, together, in inclusive educational settings. The US No Child Left Behind Act (NCLB 2002) required teaching and learning standards to be established for all students. More specifically, NCLB required that all students, (1) be included in state-wide assessments, (2) meet assessment standards, and (3) be supported by appropriate technology (including assistive technology) to achieve this. As a consequence of the re-authorised special education law of 1997, and NCLB, US systems of special education and general education no longer follow parallel but separate paths. All students, including students with disabilities, are expected to be taught, supported, and assessed in the general education environment and curriculum to the maximum extent possible.

South African inclusion initiatives, as described in the previous section, ‘Inclusive education in South Africa’, seek comparable learning models that will support the necessary adaptation in curriculum. Teachers are thus increasingly responsible for providing instruction in a way that reduces barriers and meets the needs of a growing diversity of learners. This is reflected internationally in the continued growth of inclusion initiatives in the United States, and in other countries that support equal educational access and opportunity for all learners (Brazil, Ford & Voltz 2001; Luftig & Pavri 2000; Salend 2000; Sapon-Shevin, Dobbelaere & Corrigan 1998; Zindler 2009; Peters 2004). Education systems have an increased responsibility to effectively teach learners whose learning styles and needs vary widely, through inclusive education models. Learners want and need to learn in ways that are accessible to them, and they want to have varied choices for demonstrating what they have learned. Families recognise that learners with differing needs have the right to equal opportunities to learn, and equal access to the general curriculum. Teachers, therefore, need effective models that integrate variations for learning and teaching in the goals, methods, materials, and assessments of instruction.

This goal will only be accomplished through new approaches to educational design. Universal Design for Learning (UDL) is a new model for designing all aspects of the learning environment to address the wide-ranging variation of student needs that exist in an inclusive educational system. The Center for Applied Special Technology, known as CAST Inc., first described the theory of Universal Design for Learning in 1998 (CAST 1998). Based upon brain research, and extending the architectural concept of Universal Design (Center for Universal Design 1997), the framework of Universal Design for Learning (UDL) supports these objectives, and is highly relevant for learners with widely varying needs, including learners with and without specific disabilities (Rose & Meyer 2002). Understanding and implementing UDL, therefore, can be of great interest to educators, administrators, and education support professionals around the world.

### The neurological foundation of Universal Design for Learning (UDL)

UDL is based in the fields of cognitive science and neuroscience that address the understanding of how we learn through memory, language processing, perception, problem solving, and thinking. These fields suggest that cognition involves three neural functions, (1) pattern recognition, (2) pattern planning and generation, and (3) pattern determination of importance (Rose & Strangman 2007). Lev Vygotsky and colleagues identified three essential learning components that affect levels of performance of these neural functions; (1) recognition of information to be learned, (2) application of strategies to process the information, and (3) engagement in the learning task (Vygotsky 1962). Based upon Vygotsky’s work and others, the Center for Applied Special Technology (CAST) developed the conceptual framework of UDL (Meyer & Rose 1998; Rose & Meyer 2002). The framework identifies three brain networks preferences, (1) recognition, (2) strategic, and (3) affective (Rose & Strangman 2007) which accounts for the broad diversity of learning styles and closely correlate with the work of Vygotsky (1962) and others.

### The core principles of Universal Design for Learning

The three core principles of UDL emerged from CAST’s research work on the neurological basis of learning styles, in combination with its practical work with learners (Hall, Strangman & Meyer 2003):

- **multiple means of representation:** provide multiple, flexible methods of presentation to support recognition learning (*the HOW of learning*). The teacher can present, for example, the learning materials through a variety of media (visual, auditory or tactile), and provide multiple examples that can be modified in complexity to meet a range of learning needs.
- **multiple means of action and expression:** provide multiple, flexible methods of action and expression to support strategic learning (*the WHAT of learning*). The teacher may use strategies that allow the learner to practice tasks with different levels of support and to demonstrate their knowledge and skills in a diversity of ways.
- **multiple means of engagement:** provide multiple, flexible options for engagement to support affective learning (*the WHY of learning*). This principle involves creating interesting learning opportunities that motivate and stimulate learners according to their personal backgrounds and interests.

At the heart of UDL is the design of goals, methods, materials, and assessments to make them accessible for *all* students, including those with disabilities (NCUDL 2012; Rose & Meyer 2002).

The potential impact of UDL as described by Orkwis (1999) is the:

… design of instructional materials and activities that allows learning goals to be attainable by individuals with wide differences in their abilities to see, hear, speak, move, read, write, understand English, attend, organize, engage, and remember without having to adapt thecurriculum repeatedly to meet special needs. (p. 2).

When implemented through planned curriculum design and the integrated use of supports, strategies and tools for teaching and learning, UDL holds great potential to establish truly accessible learning environments for all students. Successful implementation of UDL principles into practice does not require the use of specific technology or equipment; however, the unique capabilities offered through digital technology to transform information into accessible formats offer additional tools to use that can address learner variability. Strategic and thoughtful use of educational and assistive technologies, and appropriate strategies for their effective use, can further the implementation of UDL for many students and teachers, when used in concert with some of many other tools available to teachers that support high-quality instruction (Dalton 2005).

UDL is a conceptual and practical model for the education community, providing a framework and guidelines to change the way teachers teach, the way learners learn, and the way barriers to education for all learners can be overcome. Educators and researchers continue to develop instructional supports and strategies that will ensure successful integration of UDL in practice, informed integration of technology supports, and successful reduction of barriers to education for learners in the margins (CAST 2011; Maryland State Department of Education 2011; Meyer & Rose 2005; Paul V. Sherlock Center 2011; UDL-IRN 2012). These represent valuable resources for practitioners wanting to implement the UDL approach.

Within the UDL framework, educational planning starts with recognising and anticipating diversity in the classroom. By designing from the start, instruction and curricula that recognise, honour, and address the full range of learners’ natural variation of styles, needs, and preferences, teachers can develop, implement, and adjust a varied curriculum in which barriers to learning have been reduced or, possibly, eliminated. By employing multiple means of representation (including multisensory approaches), multiple means of student expression and actions, and multiple ways to engage and motivate learners, UDL supports maximal learning for the widest range of learners, thereby reducing the individual accommodations necessary to address specific barriers to learning arising from disability or other factors. The essence of the approach is expressed below:

UDL is designed from the outset to meet the needs of all learners, making costly, time-consuming, and after-the-fact changes unnecessary. The UDL framework encourages creating flexible designs from the start that have customizable options, which allow all learners to progress from where they are and not where we would have imagined them to be. (CAST 2011, p. 4)

This extends the possibilities for effectively including all learners in the general curriculum, and reducing the impact of barriers to learning in the educational environment (Dalton 2005). It is precisely these possibilities that the strategy of curriculum differentiation is intended to develop in South African inclusive education policy. Even with an initial understanding of the UDL framework and principles, many practical questions regarding UDL implementation still remain: What does UDL mean for a teacher in the classroom? How can a whole school develop a plan to implement UDL? What evidence exists of UDL benefit internationally? What are possible concerns or problems regarding UDL implementation? How can systems collaborate to design accessible UDL curricula?

In an effort to explore at least some of these important questions further, and recognising the potential of a ‘good fit’ between UDL and the need for curriculum differentiation skills in South African educational settings, authors were motivated to conduct a UDL workshop for South African teachers and therapists.

## Universal Design for Learning workshop at the University of Cape Town

Collaboration was established between a researcher in inclusive education at the University of Cape Town and a UDL expert who completed her postdoctoral fellowship in UDL leadership at Boston College and the CAST Centre in 2010 and who has led a UDL workgroup of university educators in Rhode Island for more than five years. This one-day workshop had the aim ‘to support teachers and therapists who are working with children with disabilities either in special schools or in the mainstream to meet a wider range of learning needs’.

The day’s programme included the following outcomes to be achieved by the end of the day:

- to understand the concept of UDL and how it can be used to ensure that all learners can learn
- to identify ways in which classroom instruction can be differentiated to meet the needs of a wide range of learners
- to understand and experience the steps involved in identifying relevant assistive devices and computer technology to support varied learning programmes.

### Participants

Invitations to the workshop were sent to teachers and therapists who work with learners experiencing barriers to learning. A total of 13 participants were involved in the workshop, representing a diverse group in terms of their professions. There were five occupational therapists, four teachers from special schools, two managers of inclusive education organisations and two speech therapists. We recognise that this is a small number of participants and it is not our intention to generalise in any way from this specific workshop experience. However, we do believe that the response of these participants can be usefully explored with a view to understanding whether UDL can meet the needs of practitioners engaged in inclusive education in South Africa to develop their skills in curriculum differentiation.

### Programme

The workshop ran for a full day and it was divided into four sessions:

- *Session 1* introduced the UDL concept to the participants, including its background, principles and its potential to improve the way the teachers and therapists promote inclusion of learners with disabilities in the learning process. Activities that explored basic UDL barriers and solutions, as identified by participants, were conducted. A brief background to inclusive education in South Africa was also presented.
- *Session 2* focused on UDL in the classroom, and addressed ways to diversify the curriculum, models for UDL application, assistive technologies (definition, scope, selection, and applications), discussion of the technology continuum and issues regarding technology in the classroom. Activities included participant discussion and identification of UDL solutions with and without technology:
- *Session 3* highlighted practical applications of UDL, use of the UDL Educator Checklist and UDL decision-making, exercises to practice checklist use as applied to video UDL case studies, debriefing, and the discussion of findings.
- *Session 4* was a concluding session where the participants had the opportunity to ask questions, and provided feedback to the presenters verbally and through the post-workshop evaluation form.

## Outcomes of the workshop

Outcomes are presented here in two ways, (1) activity results, and (2) full workshop evaluation. Activities were conducted in small groups of four or five with representation by teachers, therapists and administrators.

After a basic introduction to UDL, participants’ responses to the activity conducted in the first session were noted (Table 1). Groups were asked to respond to the statement, ‘Identify barriers that relate to the UDL Core principles and, from your own experiences, identify methods and materials that can help to address the barriers to instruction.’

**TABLE 1:** Universal Design for Learning Barriers and Solutions.

Groups	Principles					
	Principle 1: Means of representation		Principle 2: Means of action and expression		Principle 3: Multiple means of engagement	
	Barriers	Solutions	Barriers	Solutions	Barriers	Solutions
Group 1	None	FM System (sound field) with portable microphones Using diagrams via Powerpoint Multiple media Songs Movement ‘Doing’ Manipulation Use visuals in reading and visual formats (Powerpoint for visuals)	Oral presentation	Use Group discussion Enhance visuals Use a written exercise format Drawing Diagrams Flowcharts Use a written exercise format Use of colour and symbols Acting Presenting orally Experiential work (e.g. during reading) For those needing kinesthetic relievers, i.e. stress balls, pipe cleaners, rubber bands	None	Pairs for discussions Study buddy Group handling (holding spoon) Group (key points written down) Flipchart to record what people are saying Choose right learners for groups (creative groupings) Breaking up activity into smaller activity steps (task analysis) Use interests Goal focus Self-monitoring with reminder cards (visual cues)
Group 2	Standard approaches to reading & writing	Sight reading Story-telling: using pictures; on the TV	Stigma of Disability	Work on students’ strengths Teaching social and coping skills (e.g. Role playing; modelling; drama)	Parent expectation	Parent Interventions Support
Group 3	Attention Task completion Task instruction Motor output	Use a multi-sensory approach Reduce amount required in a task per ‘seating’ Consider positioning in class Consider preparation for reception (e.g. Vestibular) Use pictures Use body language	Lack of language (no receptive understanding of verbal and written) Mild intellectual and cognitive disability	Kinesthetic expression Role Play Orals Adapted technology or equipment Alternate methods of recording Eye gaze Drawing Sign Language Tactile (e.g. building, modelling)	Socio-economic (poverty, children hungry, etc.)	Shorter periods Tactile activity Give child or learner a role to play Be concrete Structuring outcomes to be more achievable Pictures Tactile (e.g. play-dough) Concrete objects (e.g. toys, action figures, etc.)

*Source*: Dalton, E.M., Instructor workshop on UDL for Disability Studies Program, University of Cape Town, South Africa, on 19 July 2011.

The second session focused on discussion of technology and how it relates to the implementation of the UDL framework, with participants’ responses to the activity conducted as illustrated (Box 1). In this activity groups were asked to respond to the statement, ‘Discuss applying UDL without technology, and with the help of technology. Identify an example for each of the 3 UDL principles. What are some of the differences, advantages, and/or disadvantages?’

Finally, the results of the full workshop evaluation indicate that all participants found the workshop to be helpful and informative and they all agreed that the information was presented at an appropriate level. Furthermore, all participants felt that they had gained a better understanding of how therapists and teachers can work together within a UDL framework and most participants (9 out of 11 respondents) felt that they were now able to identify ways in which classroom instruction can be differentiated. Fewer respondents (7 out of 11) felt that they were in a better position to choose assistive devices relevant to their learning programmes. Overall comments are included in the discussion below.

## Discussion

There is an urgent need for teachers to understand and address the range of diverse learning needs in their classrooms, if South Africa is to address the exclusion of learners from the education system. In order to do this teachers need new skills, training, and support from the educational system. Furthermore, teachers and therapists need to find ways to plan and work collaboratively, for the greatest benefit to their learners. Based on our experience with the workshop outlined in this paper, we identify several compelling reasons for using UDL as a means toward the improvement of inclusive education in South Africa. These are discussed below.

BOX 1 Universal Design for Learning Without and With Technology.

**Activity 2:**

- **Discuss the application of UDL without technology. Identify an example for each of the three UDL principles**.
- **Discuss the application of UDL with the help of technology. What are some of the differences? Advantages? Disadvantages? (Results of groups.)**

**Table:** 

**No-tech ideas**	**Technology issues and ideas**
Cooking Practical tasks Role playing Drama Worksheets Text books Constructions Models Recycled materials Group work Pictures Magazines Wall charts	Takes time to learn Technology can break down and does not always work. Social acceptability Flexibility Costs versus Creativity Documentary or Drama through video recording Use computers More interactive activities Photography
**ADHD**	**AAC technology**
Stress balls Pipe cleaners Play dough Pictures from magazines and newspapers Creative grouping Posted reminders	Board maker grid Adapted technology
**Vision**	**Vision**
Paper Blackboard Flow charts Positioning Auditory supports	Computer access Magnifying glass Dictaphone
**Representation**	**Representation**
Writing in large print Photocopy enlargement Colour overlays Manual text blocker (card, *etc*.) Acting out and Drama Bottle tops (mathematics) Magazine picture	Text enlargement Reading software Video Smartboards
**Expression**	**Expression**
Oral Written Drama, Role play Painting, Art, Poster, Craft Squeezing, Pointing (to make choices)	Speech-to-writing software Switches
**Engagement**	**Engagement**
Egg timer Handmade self-correction activity (e.g. Chart)	‘Maties’ marker timer (counts down the time left) PC program that chimes

*Source*: Dalton, E.M., Instructor Workshop on UDL for Disability Studies Program, University of Cape Town, South Africa, on 19 July 2011. ADHD, attention-deficit or hyperactivity disorder; AAC, Augmentative and Alternative Communication; UDL, Universal Design for Learning.

UDL is, as its name suggests, an attempt to maximise learning in a universal manner. As such, it aims to apply the same principles to *all* learning rather than proposing specific learning programmes for different forms of diversity or disability. This allows for a certain simplicity that is very attractive to the busy teacher. If (s)he can implement the basic principles by planning for a variety of presentation methods, allowing for different forms of expression and engaging learners emotionally, then a whole range of needs can be met.

An additional advantage of UDL is that therapists and teachers can readily share the language of UDL. Whereas teachers speak the language of the curriculum, therapists are more steeped in medical or psychological terms. By paring down teaching and learning to the three processes of flexible methods of presentation, expression and engagement, all those working with the learner can collaborate with a common understanding. Participant suggestions as to how UDL can be implemented, has been noted (Table 1). As one participant commented, ‘There was lots of room to apply and define more specific ways for therapists and teachers to work together’ (Occupational therapist 1).

Participants generally benefitted from the practical nature of the workshop, as indicated by the overall workshop evaluation results; however, a one-day workshop was limited by time and could not cover all areas as originally anticipated. Some comments revealed that participants ‘would have wanted more time to practice the checklist’, and ‘this area (UDL and transition) was not explored in depth’. Future workshops should take these comments into consideration, and plan for extended sessions that would include several practice sessions with UDL implementations tools to help participants increase their competence and confidence in UDL implementation.

The implementation of UDL is often regarded as a high-technology option; however, learning activities conducted in the workshop showed that technology can be pursued at many different levels (Box 1). Smart-boards, I-Pads and other tools can contribute to achieving educational outcomes, but low-tech options can achieve the similar outcomes when implemented by using the three core UDL principles. It was in this connection that a discussion took place on how an education recycling centre that focuses on useful teaching materials could be set up for teachers as a resource for the further implementation on UDL in South Africa. Overall, participants found UDL relevant to the South African situation: ‘The concepts were applicable to the South African context … this will need support from the department of education’ (Special education teacher 2).

As a systems change initiative, UDL offers a framework that supports the design of instruction that integrates many variables and variations of learners’ and educators’ needs, but the potential for change cannot be realised without significant and on-going training and professional development of all professionals involved in the system of education. Participants called for continued instruction in UDL and inclusive education. Some comments included, ‘session was fruitful, hope for more of these in the future’, and ‘course extremely relevant to current interests’. Such instruction would be beneficial not only for teachers and therapists, but also for administrators of educational systems.

Many questions about UDL and its implementation in classrooms and educational systems in the USA and around the world remain to be addressed. Some questions were identified earlier in this paper, and others have emerged from the field (Edyburn 2010).

Organisations such as the UDL Implementation and Research Network (UDL-IRN, http://udl-irn.org/) are focusing on such questions and striving to develop tools and resources to address them. The Center for Applied Special Technology (CAST, www.cast.org) and the National UDL Center (www.udlcenter.org) continue their efforts to develop UDL as a comprehensive curriculum design framework and approach that can effectively support the inclusion of *all learners* in the general education curriculum.

With one day of training in UDL, workshop participants were able to identify examples relating to the three UDL core principles, example of barriers to UDL implementation, ways to implement UDL with and without technology, and started to explore the use of an educator checklist tool for UDL analysis and planning. This testifies to the attractive simplicity of the method. In consideration of the enthusiasm with which the participants received the UDL concepts, the authors (who were the organisers and presenters of the workshop) are exploring ways by which workshops of this nature can continue in South Africa to promote the implementation of UDL in South African Schools. The authors see this as an avenue that will enhance inclusion of learners who experience barriers to learning in South Africa and promote effective transition from school to productive work.

## Ethical considerations

All participants attended the workshop voluntarily and consented to the use of their evaluation forms and activity notes for course development, including research. There was no risk of harm to the participants and their anonymity was maintained.
